# A novel miR-7156-3p-HOXD13 axis modulates glioma progression by regulating tumor cell stemness

**DOI:** 10.7150/ijbs.51293

**Published:** 2020-10-21

**Authors:** Junfeng Zhang, Mengsheng Deng, Haipeng Tong, Wei Xue, Yu Guo, Jianmin Wang, Lizhao Chen, Shunan Wang

**Affiliations:** 1 Department of Radiology, Daping Hospital, Army Medical University, Chongqing 400042, China.; 2 State Key Laboratory of Trauma, Burn and Combined Injury, Daping Hospital, Army Medical University, Chongqing 400042, China.; 3 Chongqing Clinical Research Center of Imaging and Nuclear Medicine, Chongqing 400042, China.; 4 Department of Neurosurgery, Daping Hospital, Army Medical University, Chongqing 400042, China.

**Keywords:** miR-7156-3p, HOXD13, stemness, glioma, invasion, biomarker

## Abstract

Malignant glioma is the most common brain tumor in adults. Despite the great advances in anti-glioma treatments which have led to significant improvement in clinical outcomes, tumor recurrence remains the major cause of mortality. Increased cancer cell stemness and invasiveness are correlated with glioma progression. By searching the Cancer Genome Atlas, we showed that the expression of miR-7156-3p is significantly decreased in glioma tissues compared to the normal brain, and the decreased level of miR-7156-3p is closely correlated with glioma grade and patient survival. Clinical study consistently confirmed that miR-7156-3p is negatively correlated with glioma grade. Cell culture and animal experiments revealed that inhibition of miR-7156-3p effectively stimulates glioma cell stemness, invasion, and growth. In contrast, the augmentation of miR-7156-3p inhibits these phenotypes. Using Next-generation sequencing combined with target prediction approach, Homeobox D13 (HOXD13) is identified as the target gene of miR-7156-3p and further validated by luciferase reporter assay and cell transfection experiments. Additional *in vitro* and animal experiments demonstrated that miR-7156-3p regulates glioma cell stemness, invasion, and growth by mediating HOXD13. In conclusion, our findings provide new insight into the regulation of glioma stemness and invasiveness and may propose a potential strategy for anti-glioma treatment. Moreover, miR-7156-3p may serve as a candidate biomarker for predicting glioma progression in clinical practice.

## Introduction

Malignant glioma is the most common primary malignancy in the brain, accounting for 80% of all brain tumors 1. Surgical resection adjuvant with chemoradiotherapy is the standard therapeutic regimen for glioma. However, patients with glioma often show a high rate of recurrence after treatment 2. Even with the great advances in emerging surgical technology and therapeutic strategy, malignant glioma often recurs at the region within 1-2 cm of the original tumor site due to the tumor cells invading into the surrounding normal brain tissue where they can hide from surgical removal and chemoradiotherapy, suggesting that glioma cell invasiveness plays a critical role in tumor progression 3. Previous studies have reported that cancer stem cells (CSCs) are the initialing factor responsible for glioma recurrence. CSCs are a small subpopulation of cancer cells that have high invasiveness and self-renewal ability to differentiate into tumor cells 4, 5. Therefore, glioma invasion and stemness are a lethal combination, resulting in tumor progression and therapeutic resistance 6. These findings indicate that inhibiting glioma cell invasion by specifically targeting CSCs is important with significant clinical implications for anti-glioma treatment. Unfortunately, the mechanisms of glioma cell invasion and regulation of CSCs are not fully understood and no effective treatment aiming at glioma cell invasion and CSCs exists thus far.

MicroRNAs (miRNAs) are a class of small non-coding single-stranded RNAs that negatively regulate gene expression by stimulating the degradation of or inhibiting the translation of mRNAs by binding to 3'-untranslated region (3`-UTR) of the target genes. Dysregulated expression of miRNAs is identified in most cancer types including glioma 7. It is well-documented that certain aberrantly expressed miRNAs are closely associated with glioma recurrence 8, stimulation of glioma cell stemness 9, and tumor invasiveness 10. More importantly, inhibition or overexpression of miRNAs that are aberrantly upregulated 11 or downregulated 12, 13 in glioma significantly inhibits tumor growth and invasion, indicating that miRNAs have much potential as promising potential therapeutic targets. However, some miRNAs play opposite roles by targeting different target genes in different types of cancers, even in the same cancer type but at different grades or stages. For example, Korpal et al. 14 found that miR-200s inhibit local invasion by targeting ZEB1 but stimulate metastatic colonization by targeting Sec23a in breast cancer. Also, studies show that even aberrant expression of a single miRNA is sufficient to cause cancer progression, suggesting the essential role of miRNA in tumorigenesis 15. Therefore, studying individual miRNA that abnormally expressed in tumors is necessary to fully understand its functional roles in glioma and is the prerequisite for clinical translation of miRNA-targeted therapy.

In this study, we reported for the first time that miR-7156-3p is significantly downregulated in glioma and closely associated with glioma grade, invasion, stemness, and survival in glioma patients. Moreover, we unveiled that miR-7156-3p overexpression dramatically suppressed glioma stemness, invasiveness, and tumor growth by downregulating HOXD13.

## Materials and Methods

### Cell culture and tumor specimens

Human glioma cell lines (U373 and SW1783) were obtained from ATCC and cultured in MEM (Gibco) with 10% fetal bovine serum (FBS, Invitrogen). Formaldehyde-fixed, paraffin-embedded (FFPE) tumor specimens from 44 newly-diagnosed glioma patients underwent tumor resection at Daping Hospital of Army Medical University were used in the study. The characteristics of the patients were summarized in Table 1. The study was approved by the Daping Hospital review boards.

### mRNA analysis

Total RNA from tumor cells and fresh tissues was isolated using TRIzol reagent (Invitrogen) following the manufacturer's instruction. Total RNA from FFPE tumor specimens was isolated using the RNA Prep Pure FFPE Kit (QIAGEN, Hilden, Germany). Reverse transcription was performed using a High-Capacity cDNA Reverse Transcription Kit (Applied Biosystems, Foster City, CA, USA). Mature miR-7156-3p and the U6 small nuclear RNA (RNU6) endogenous control were analyzed by PCR with a specific primer set and kit from RiboBio (Guangzhou, China). The primers used for HOXD13 amplification were 5'-CGCTGCCTCTGGCAAGTGGAGT-3' and 5'-TCGGTTATGGTACAAAGCGGAGAC-3'. The primers used for GAPDH amplification were 5'-GGAGCGAGATCCCTCCAAAAT-3' and 5'-GGCTGTTGTCATACTTCTCATGG-3'.

### Cell Counting Kit-8 analysis

Glioma cells were transfected with the indicated oligonucleotides and plasmids. After 24 hours of transfection, cells were reseeded into 96-well cell culture plates at a density of 1×10^4^ cells per well, and cell viability was measured using a Cell Counting Kit-8 (CCK-8, Wuhan, China) assay according to the manufacturer's protocol at the indicated times.

### Luciferase reporter assay

A luciferase reporter assay was performed as described previously 16. In brief, the 3`-UTR fragments of HOXD13 predicted to interact with miR-7156-3p were amplified by PCR from human genomic DNA and inserted into the *Mlu* І and *Hind* III sites of the pMIR-REPORTTM Luciferase vector (Thermo Fisher Scientific). Then, this firefly luciferase plasmid and Renilla luciferase plasmid were cotransfected into tumor cells. After 48 hours of transfection, the luciferase activity was examined using a Dual-Luciferase Assay System (Promega).

### Apoptosis analysis

Glioma cells were transfected with the indicated oligonucleotides and plasmids. After 48 hours of transfection, cells were harvested and stained with annexin V and 7-aminoactinomycin D (7-AAD), and then subjected to flow cytometric analysis.

### Invasion assay

The invasion assay was performed as described previously 16. The indicated glioma cells were transfected with the indicated oligonucleotides and plasmids. After 48 hours, 1×10^4^ cells in serum-free growth medium were seeded in the upper wells of transwell chambers. The lower wells contained the same medium supplemented with 10% FBS. After 24 hours of culture, the cells on the upper side of the chamber were removed, and the cells on the lower side of the chamber were fixed with glutaraldehyde, stained with 0.1% crystal violet, and counted.

### Western blotting and immunohistochemistry

Western blotting and immunohistochemistry (IHC) were performed as described previously 17. Antibodies against HOXD13, CD133, CD44, OCT4, SOX2, and β-Actin were purchased from Abcam.

### Animal experiments

Male BALB/c nude mice (6-8week, 20-25 g, obtained from the Experimental Animal Center of Daping Hospital, Chongqing, China) were used for xenograft modeling. Briefly, 1×10^6^ U373 cells suspended in 10 μL DPBS were stereotactically implanted into the right cerebral cortex of mice using the Hamilton syringe. All the surgical procedures and handling of the animals were performed in accordance with the International Principles of Laboratory Animal Care and were approved by the Animal Use Subcommittee of Daping Hospital, Army Medical University.

Assessments of tumor volumes were performed with a 7T animal magnetic resonance imaging (MRI) scanner (Biospin70/20, Bruker, Ettlingen, Germany) equipped with a four-channel mouse head transmit/receive coil. *T*_2_-weighted images (Turbo-RARE; TE/TR: 45ms/4000ms, field of view: 25×25 mm, matrix size: 256×256, slice thickness: 0.5 mm) were conducted to evaluate tumor volumes according to the formula: [width]^2^ × [length] × ^1^/_2_.

### Statistical analysis

The differences between treatment groups were analyzed with one-way ANOVA using SAS software (SAS Institute Inc., Cary, NC, USA). All data were presented as the mean ± standard deviation (SD). The correlations between gene expression and glioma grade were determined using Pearson's chi-squared test. The overall survival (OS) of patients was calculated using the Kaplan-Meier survival curve. The results were considered significant only when *P* < 0.05.

## Results

### miR-7156-3p is downregulated in glioma and is associated with glioma progression

**Figure 1:**
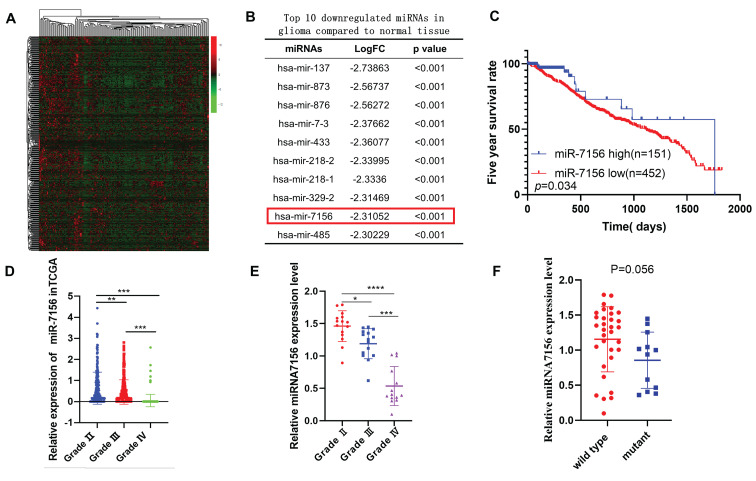
** The decreased expression level of miR-7156-3p in glioma significantly correlates with glioma grade and survival rate of glioma patients. A.** Heat map of differentially expressed miRNAs between grade II glioma (n=152) and normal brain tissues (n=5). Data were obtained from the TCGA database. **B.** List of the top 10 downregulated miRNAs in glioma tissues compared to normal brain tissues. Data were obtained from the TCGA database. **C.** Kaplan-Meier analysis of the overall survival rate of patients with glioma with low and high miR-7156-3p expression. Data were obtained from the TCGA database. **D.** miR-7156-3p expression levels are negatively correlated with glioma grade. Data were obtained from the TCGA database. Grade II, n=249; grade III, n=261; grade IV, n=174. **E.** Our clinical cohort analysis showed that miR-7156-3p expression levels are negatively correlated with glioma grade. The expression level of miR-7156-3p was measured using qRT-PCR. **F.** Our clinical cohort analysis showed that IDH-1 mutation did not affect miR-7156-3p expression in glioma. *, *P*<0.05. **, *P*<0.01; ***, *P*<0.001.

To identify glioma-associated miRNAs, we analyzed the Cancer Genome Atlas (TCGA) dataset and identified global miRNAs that were significantly dysregulated in glioma compared to normal brain tissues (Figure 1A). Among the top 10 miRNAs with the largest fold change (Figure 1B), the role of other miRNAs in glioma has been reported except miR-7156-3p 18-26. Therefore, we focused on investigating the role of miR-7156-3p in glioma. Clinical data analysis showed that low expression level of miR-7156-3p was closely correlated with poor prognosis in glioma patients (Figure 1C). More importantly, we observed a negative correlation between miR-7156-3p expression level and glioma grade in both TCGA dataset (Figure 1D) and our clinical cohort data (Figure 1E), indicating that miR-7156-3p has important clinical significance in glioma. However, no significant miR-7156-3p difference was seen between glioma with or without isocitrate dehydrogenase-1 mutation (IDH-1) (Figure 1F). Taken together, these data suggest that decreased expression of miR-7156-3p may be involved in the glioma progression and patient survival independent of IDH-1 mutation.

### miR-7156-3p negatively regulates glioma progression

**Figure 2:**
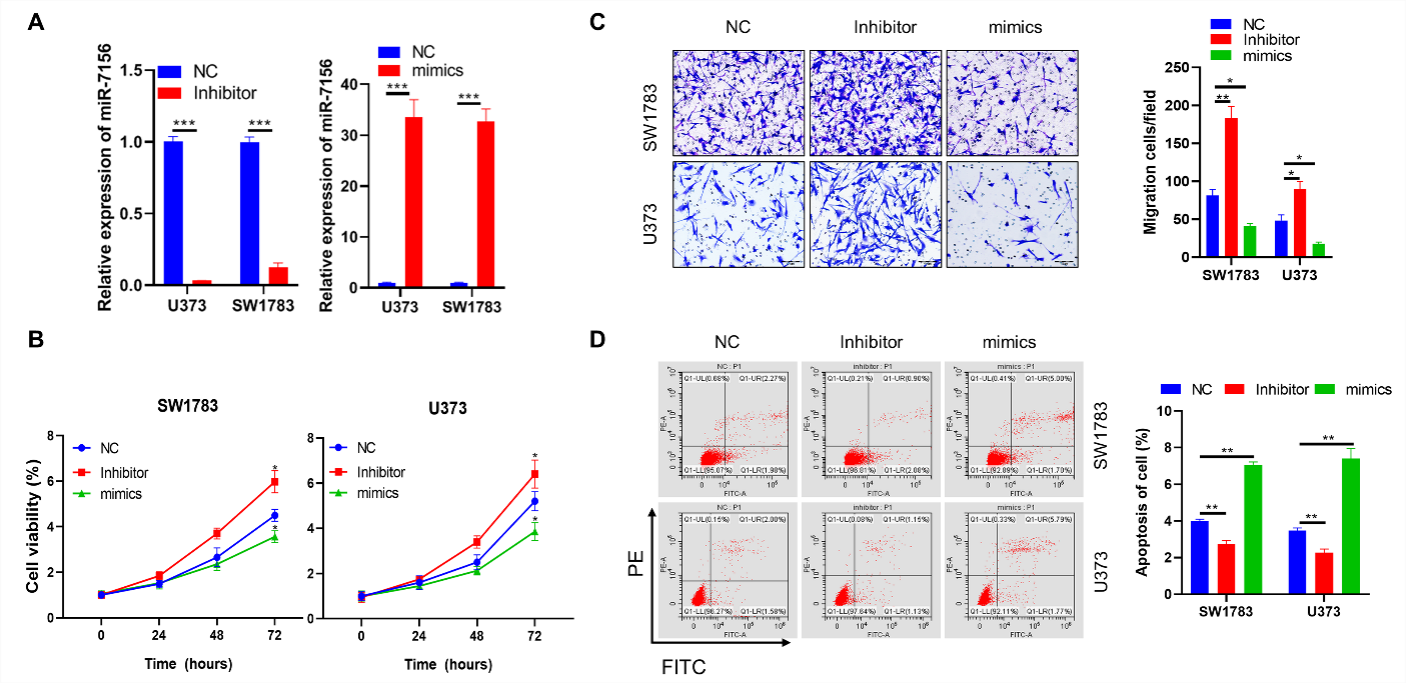
** miR-7156-3p negatively regulates glioma progression. A.** miR-7156-3p expression levels were measured in glioma cell lines. The indicated cells were transfected with the indicated oligonucleotides and incubated for 72 hours. Then, the cells were subjected to qRT-PCR analysis. **B.** miR-7156-3p negatively regulated glioma cell growth. The indicated cells were transfected with the indicated oligonucleotides and incubated for 24 hours. Then, the cells were subjected to cell growth analysis. **C.** miR-7156-3p negatively regulated glioma cell invasion. The indicated cells were transfected with the indicated oligonucleotides and incubated for 48 hours. Then, the cells were subjected to an invasion assay. **D.** miR-7156-3p stimulated glioma cell apoptosis. The indicated cells were transfected with the indicated oligonucleotides and incubated for 48 hours. Then, the cells were subjected to apoptotic cell analysis. NC, negative control oligonucleotides; mimics, miR-7156-3p mimics; inhibitor, inhibitor of miR-7156-3p, ***, *P*<0.001.

To investigate whether the decreased expression of miR-7156-3p directly contributes to glioma progression, both U373 and SW1783 glioma cells were used and transfected with miR-7156-3p inhibitor and then subjected to *in vitro* functional analysis. Our data showed that inhibition of miR-7156-3p (Figure 2A) significantly increased cell growth (Figure 2B), invasion (Figure 2C), and suppressed apoptosis (Figure 2D) in both SW1783 and U373 cells. In contrast, overexpression of miR-7156-3p (Figure 2A) dramatically inhibited cell growth (Figure 2B), invasion (Figure 2C), and induced cell apoptosis (Figure 2D). These data indicate that inhibition of miR-7156-3p stimulates glioma progression and that upregulating miR-7156-3p may serve as a potential strategy for anti-glioma treatment.

### miR-7156-3p negatively regulates glioma stemness

**Figure 3:**
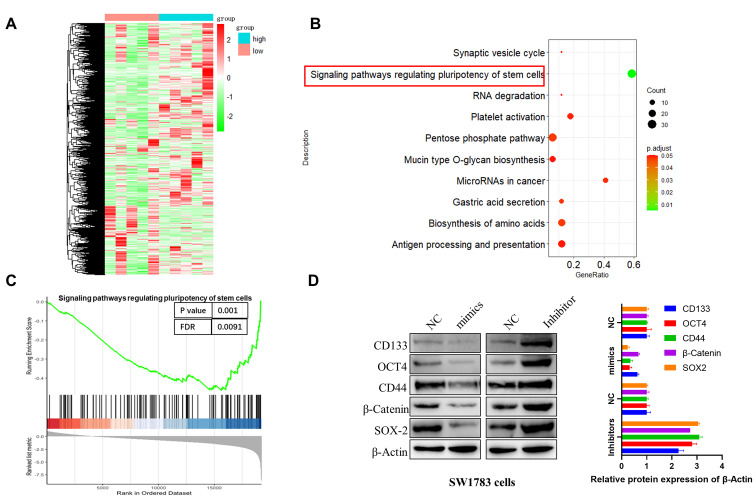
** miR-7156-3p negatively regulates glioma stemness. A.** Heat map of differentially expressed genes between miR-7156-3p high-expressing glioma specimens (n=5) and miR-7156-3p low-expressing glioma specimens (n=5). **B-C.** Pathway enrichment and GSEA analysis was performed using the mRNA sequencing data. **D.** miR-7156-3p negatively regulated glioma cell stemness. The indicated cells were transfected with the indicated plasmids. After 72 hours of transfection, cancer stem cell biomarker expression was detected by Western blotting.

To investigate the molecular mechanism of miR-7156-3p in the glioma progression, we performed mRNA sequencing using glioma specimens with high or low miR-7156-3p expression (Figure 3A) and conducted pathway enrichment and gene set enrichment analysis (GSEA). As shown in figures 3B and 3C, miR-7156-3p is closely involved in the regulation of stem cell-related signaling pathways. This result was further confirmed by the detection of stem cell marker proteins. Western blotting analysis showed that overexpression of miR-7156-3p significantly suppressed while silencing of miR-7156-3p increased the expression of stem cell marker proteins in SW1783 cells (Figure 3D). Overall these findings suggest that miR-7156-3p negatively regulates glioma cell stemness.

### HOXD13 is a target of miR-7156-3p in glioma

**Figure 4:**
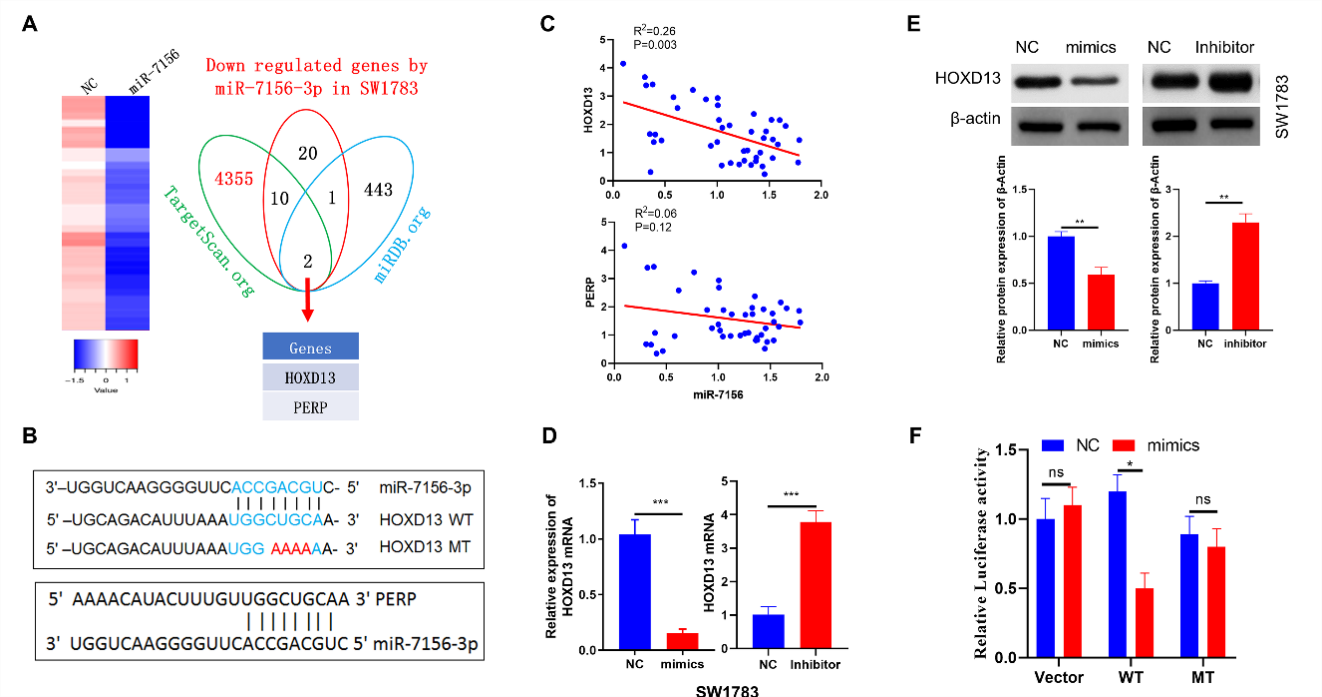
** HOXD13 is a target gene of miR-7156-3p in glioma. A.** Candidate targets of miR-7156-3p in glioma cells. mRNA sequencing identified 33 genes (heat map) that were significantly downregulated in miR-7156-3p-overexpressing U373 cells relative to control cells. Then, through a search of the miRNA databases TargetScan and miRDB, two candidate target genes of miR-7156-3p were determined. **B.** Predicted binding sites of miR-7156-3p in the 3`-UTRs of PERP and HOXD13. Mutations in the 3`-UTR of HOXD13 are highlighted in red. **C.** Correlation between miR-7156-3p expression level and HOXD13 and PERP expression levels in glioma. Data from the TCGA database. **D.** HOXD13 mRNA expression levels were measured in the indicated glioma cells. Cells were transfected with mimics or inhibitor of miR-7156-3p. After 72 hours of transfection, cells were subjected to qRT-PCR analysis. **E.** miR-7156-3p inhibited HOXD13 protein expression in SW1783 cells. Cells were transfected with mimics or inhibitor of miR-7156-3p. After 72 hours of transfection, the cells were subjected to Western blotting analysis. **F.** Luciferase activity of the reporter driven by the wild-type or mutant 3`-UTR of HOXD13 in U373 cells cotransfected with control oligonucleotides (NC) or miR-7156-3p mimics. A luciferase reporter containing the wild-type or mutant 3`-UTR of HOXD13 was constructed and transfected into U373 cells with or without miR-7156-3p mimics. After 48 hours of transfection, luciferase intensity was assessed. ***P < 0.001; ns, not significant.

miRNAs play an important role in the regulation of gene expression by inhibiting target gene translation. Thus, to identify the target genes of miR-7156-3p in glioma, we performed next-generation sequencing using miR-7156-3p-overexpressing U373 glioma cells and their control cells. As shown in figure 4A, we detected a total of 33 genes that were significantly downregulated in miR-7156-3p-overexpressing cells compared to the control. By applying these genes to miRNA target prediction database searches (TargetScan.org and miRDB.org), it revealed that the 3'-UTRs of HOXD13 and PERP sequences contained potential miR-7156-3p binding site (Figures 4A and 4B). Next, we used the TCGA dataset to investigate the correlations between miR-7156-3p expression levels and HOXD13 or PERP expression levels in glioma. TCGA dataset analysis showed that the miR-7156-3p expression level was negatively correlated with the HOXD13 expression level in glioma but not with the PERP expression level (Figure 4C), suggesting that HOXD13 may be a major target of miR-7156-3p in glioma. To determine whether miR-7156-3p is directly involved in the regulation of HOXD13 expression, miR-7156-3p mimics or inhibitors were transfected into SW1783 cells (Figure 2A) and the HOXD13 expression levels were measured. Our data showed that overexpression of miR-7156-3p significantly inhibited HOXD13 expression, while inhibition of miR-7156-3p increased HOXD13 expression in glioma cells both at the mRNA (Figure 4D) and protein levels (Figure 4E). Furthermore, we investigated the direct interaction between miR-7156-3p and the 3'-UTR of HOXD13 using a luciferase reporter assay. The luciferase assay results showed that miR-7156-3p significantly inhibited luciferase activity in glioma cells transfected with the wild-type 3'-UTR of HOXD13 but not in these transfected with the mutant 3'-UTR of HOXD13 (Figure 4F). Taken together, our findings indicate that HOXD13 is a target of miR-7156-3p in glioma and that miR-7156-3p suppresses HOXD13 expression by directly binding to its 3'-UTR.

### HOXD13 stimulates glioma stemness and progression

**Figure 5:**
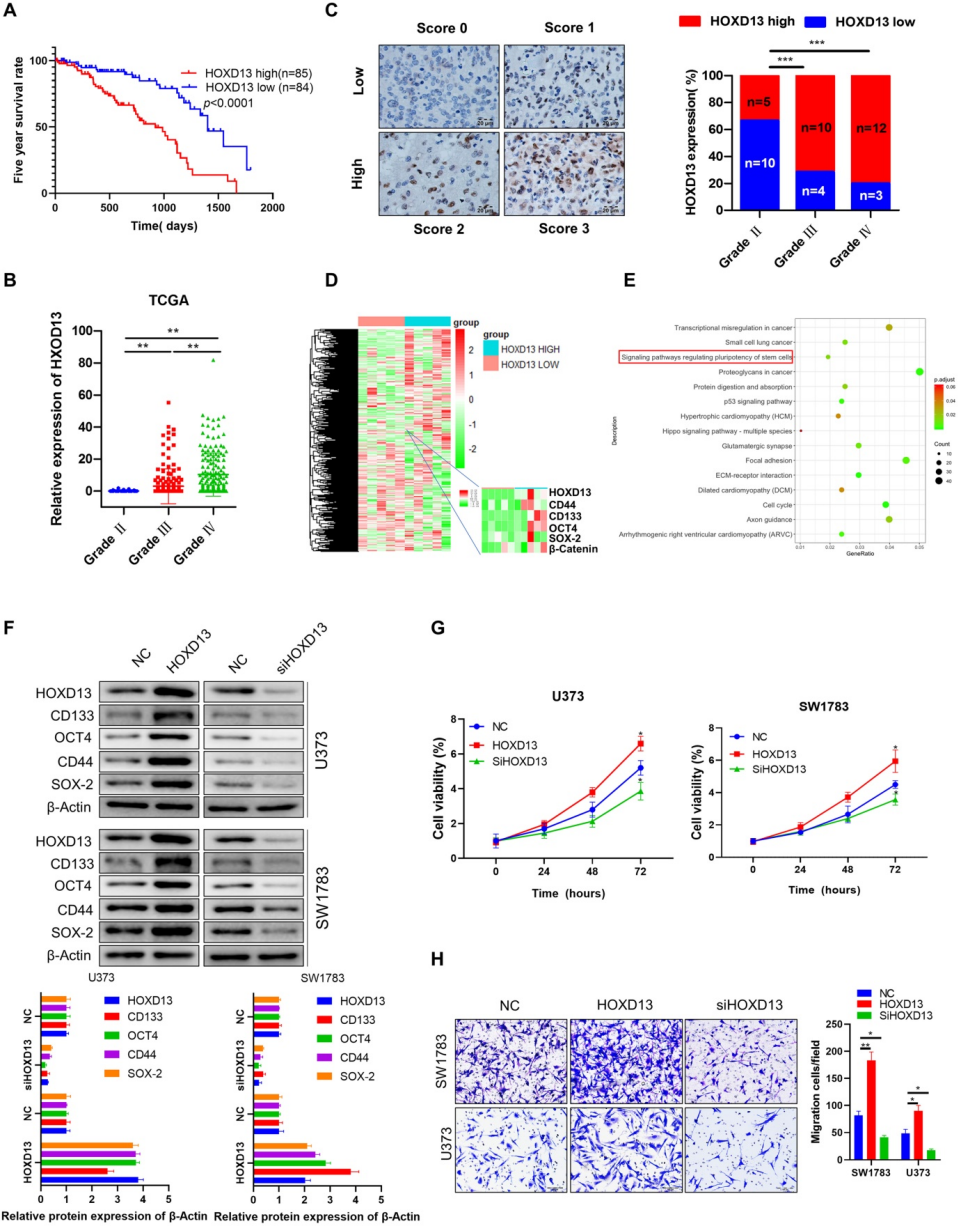
** HOXD13 positively regulates glioma stemness and progression. A.** Kaplan-Meier analysis of the overall survival rate of patients with glioma with low and high HOXD13 expression. Data were obtained from the TCGA database. **B.** TCGA dataset analysis showed that the expression level of HOXD13 positively correlated with glioma grade. Grade II, n=249; grade III, n=261; grade IV, n=174. **C.** Our cohort samples analysis showed that the HOXD13 expression level is correlated with glioma grade. The expression level of HOXD13 was measured by IHC. **D.** Heat map of differentially expressed genes between HOXD13 high-expressing glioma specimens (n=5) and HOXD13 low-expressing glioma specimens (n=5). **E.** Pathway enrichment analysis was performed using the mRNA sequencing results (data from Figure 5D). **F.** HOXD13 positively regulated glioma cell stemness. The indicated cells were transfected with the indicated plasmids. After 72 hours of transfection, cancer stem cell biomarker expression was detected. **G.** HOXD13 positively regulates glioma cell growth. The indicated glioma cells were transfected with the indicated plasmid, and cell viability was measured at the indicated times. **H.** HOXD13 positively regulates glioma cell invasion. The indicated glioma cells were transfected with the indicated plasmids. After 48 hours of transfection, the cells were subjected to an invasion assay.

The role of HOXD13 in glioma is an uncharted territory currently. Therefore, we investigated the function of HOXD13 in glioma. The results of Kaplan-Meier analysis using TCGA dataset showed that a high expression level of HOXD13 was closely associated with a lower survival rate in glioma patients (Figure 5A). Moreover, the mRNA expression level of HOXD13 was closely correlated with glioma grade (Figure 5B). In accord with this result, our clinical cohort also showed that HOXD13 expression was positively correlated with glioma grade (Figure 5C). To further investigate the role of HOXD13 in glioma, we performed mRNA sequencing using glioma specimens with high or low HOXD13 expression (Figure 5D). Pathway enrichment analysis showed that HOXD13 was involved in the regulation of stem cell-related signaling pathways (Figure 5E). To further validate this finding, western blotting was performed showing that HOXD13 overexpression significantly increased, while silencing of HOXD13 decreased the expression of stem cell marker proteins in glioma cells (Figure 5F). Similarly, CCK-8 and invasion assays showed that HOXD13 positively regulated glioma cell growth (Figure 5G) and invasion (Figure 5H). These findings suggest that HOXD13 plays an oncogenic role in glioma and it may be a potential therapeutic target.

### miR-7156-3p plays an anti-tumor role by mediating HOXD13 in glioma

**Figure 6:**
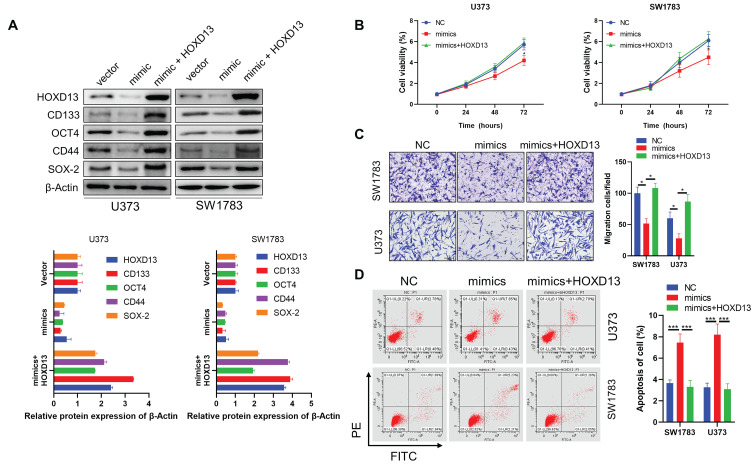
** miR-7156-3p anticancer effects in glioma cells are attenuated by HOXD13 overexpression. A.** Stemness inhibition by miR-7156-3p was attenuated by HOXD13 overexpression in glioma cells. The indicated cells were transfected with the indicated oligonucleotides and/or plasmids. After 72 hours, the cells were subjected to Western blotting. **B.** Invasion inhibition by miR-7156-3p was attenuated by HOXD13 overexpression in glioma cells. The indicated cells were transfected with the indicated oligonucleotides and/or plasmids. After 48 hours, the cells were subjected to an invasion assay. **C.** Growth inhibition by miR-7156-3p was attenuated by HOXD13 overexpression in glioma cells. The indicated cells were transfected with the indicated oligonucleotides and/or plasmids. Cell viability was measured using a CCK-8 kit. **D.** Apoptosis stimulation of miR-7156-3p was attenuated by HOXD13 overexpression in glioma cells. The indicated cells were transfected with the indicated oligonucleotides and/or plasmids. After 48 hours of transfection, cells were subjected to flow cytometry analysis.

Next, we investigated whether HOXD13 is involved in the anticancer role of miR-7156-3p in glioma. Our data showed that overexpression of miR-7156-3p induced anti-tumor effects that were significantly attenuated by HOXD13 overexpression in glioma cells, including tumor cell stemness (Figure 6A), invasion (Figure 6B), growth (Figure 6C), and apoptosis (Figure 6D). These findings demonstrate that miR-7156-3p plays an anti-tumor role in glioma by mediating HOXD13 expression.

### Overexpression of miR-7156-3p significantly inhibits glioma progression *in vivo*

**Figure 7:**
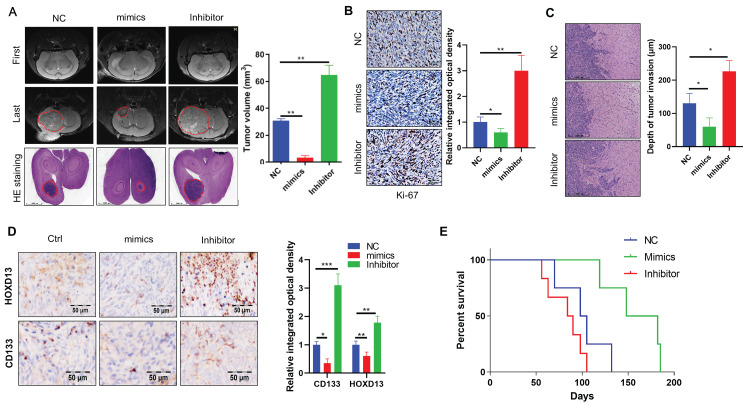
** miR-7156-3p inhibits glioma progression in an animal model. A.** miR-7156-3p negatively regulates glioma growth in an animal model. Tumor growth was detected using MRI. First, MR images at start time; Last, MR images after one month of tumor implantation. **B.** Ki-67-positive cells were detected by IHC in glioma tissues from animal models. **C.** Invasiveness was assessed using the distance between the tumor mass edge and the invasive lesion. **D.** HOXD13 expression level and CD133-positive glioma cells were detected using IHC in glioma tissues from animal models. **E.** miR-7156-3p positively correlated with survival time in xenograft models (n=6 per group).

Finally, we established orthotopic xenograft models to confirm the effect of miR-7156-3p on glioma progression* in vivo*. Orthotopic glioma animal models were generated using U373 cells expressing miR-7156-3p or antisense miR-7156-3p. MRI scanning and HE staining showed that inhibition of miR-7156-3p significantly stimulated glioma growth, while overexpression of miR-7156-3p dramatically inhibited glioma growth (Figure 7A). IHC results of the cell proliferation marker protein Ki-67 showed that inhibition of miR-7156-3p enhanced glioma cell proliferation, but not in tumor tissues with overexpression of miR-7156-3p (Figure 7B). Additionally, we evaluated the tumor invasion depth from the border of the tumor mass to the invaded cells. The depth of tumor invasion was remarkably increased by miR-7156-3p inhibition but decreased when miR-7156-3p was overexpressed (Figure 7C). Moreover, histologic analysis showed that levels of HOXD13 and CD133 reduced when miR-7156-3p levels were augmented but increased when miR-7156-3p was inhibited (Figure 7D). Notably, compared with the control group, inhibition of miR-7156-3p shortened the survival time of glioma xenograft model mouse, while overexpression of miR-7156-3p significantly prolonged the survival time (Figure 7E). Collectively, these findings indicate that the decreased levels of miR-7156-3p contribute to glioma progression *in vivo* and that overexpression of miR-7156-3p may be a promising strategy for anti-glioma treatment.

## Discussion

Accumulating studies show that aberrant expression of miRNAs is associated with most cancer types, including glioma 10, 27. Here, we first report that decreased expression levels of miR-7156-3p in glioma are closely associated with poor clinical outcome in glioma patients. Our *in vitro* and *in vivo* experiments demonstrate that inhibition of miR-7156-3p enhances glioma stemness, growth and invasion. Importantly, overexpression of miR-7156-3p significantly inhibits glioma stemness, growth, invasiveness and induce glioma cell apoptosis. Our findings also indicate that the level of miR-7156-3p is associated with glioma grade and patient survival. In this regard, miR-7156-3p may have much potential to serve as a prognostic candidate biomarker. Additionally, overexpression of miR-7156-3p may be a useful strategy for anti-glioma therapy.

We next investigated the functional mechanism of miR-7156-3p in glioma. MiRNAs play a pivotal role by inhibiting target gene expression. In this work, we identify and validate that HOXD13 is the predominant target of miR-7156-3p in glioma by using a series of experiments. Our transcriptome analysis, qRT-PCR, and Western blotting analysis show that miR-7156-3p negatively regulates HOXD13 expression at both the mRNA and protein levels in glioma cells. Similarly, clinical data from the TCGA database shows that the expression levels of miR-7156-3p and HOXD13 are negatively correlated in glioma. Furthermore, the luciferase assay shows that miR-7156-3p inhibits HOXD13 expression by directly binding to the 3-UTR of HOXD13. Moreover, functional experiments demonstrate that the miR-7156-3p-induced anti-glioma effects are significantly attenuated by overexpression of HOXD13. These results indicate that miR-7156-3p plays its anti-tumor role through HOXD13 inhibition.

Upregulated expression of HOXD13 in glioma is previously identified, but the function of HOXD13 in glioma remains unclear. Clinical data analysis from the TCGA database showed that a high level of HOXD13 was closely associated with glioma grade and a low survival rate in glioma patients. Our experiments suggest that this association may be a functional result of HOXD13 in stimulating glioma cell stemness, invasion, and growth. These data demonstrate that HOXD13 plays an oncogenic role in glioma, but its specific mechanism in glioma needs further study.

In conclusion, our study determines that miR-7156-3p acts as a tumor suppressor in glioma by targeting HOXD13. Moreover, a decreased expression level of miR-7156-3p is a prognostic marker candidate in glioma patients, and miR-7156-3p may be a promising therapeutic target by inhibiting tumor cell stemness for anti-glioma treatment.

## Figures and Tables

**Table 1:** Characteristics of glioma patients

Characteristics	Variable	Number
Age (years)	Range (means±SD)	16-78 (60±12)
Gender	Male	23 (52.3)
	Female	21 (47.7)
Family history	No	31 (70.5)
	Yes	13 (29.5)
Grade	II	14 (31.8)
	III	15 (34.1)
	IV	15 (34.1)
IDH-1	Wild type	32 (72.7)
	Mutant	12 (27.3)

## Abbreviations

HOXD13: Homeobox D13
CSCs: cancer stem cells
miRNAs: MicroRNAs
3`-UTR: 3`-untranslated region
FFPE: formaldehyde-fixed paraffin-embedded
FBS: fetal bovine serum
CCK-8: Cell Counting Kit-8
7-AAD: 7-aminoactinomycin D
IHC: immunohistochemistry
SD: standard deviation
OS: overall survival
TCGA: the Cancer Genome Atlas
GSEA: gene set enrichment analysis
IDH-1: isocitrate dehydrogenase-1
